# Early Staged STABILISE Technique Achieves Favorable Aortic Remodeling after Central Repair for Acute Type A Aortic Dissection

**DOI:** 10.3400/avd.cr.26-00132

**Published:** 2026-10-28

**Authors:** Sho Takagi, Yoshihiro Goto, Masahiro Inagaki, Makoto Taniwaki, Atsuki Imagawa, Yasuhide Okawa

**Affiliations:** Department of Cardiovascular Surgery, Toyohashi Heart Center, Toyohashi, Aichi, Japan

**Keywords:** acute type A aortic dissection, residual distal aortic dissection, stent-assisted balloon-induced intimal disruption and relamination in aortic dissection repair (STABILISE)

## Abstract

Residual distal aortic dissection often persists after repair of acute type A aortic dissection, and may cause progressive aortic enlargement requiring secondary intervention. We describe a 43-year-old male who underwent an early staged stent-assisted balloon-induced intimal disruption and relamination in aortic dissection repair (STABILISE)-thoracic endovascular aortic repair (TEVAR) procedure after central repair for an acute type A aortic dissection. Early postoperative computed tomography angiography demonstrated persistent false lumen perfusion, progressive false lumen enlargement, and marked true lumen compression. Thirty-eight days postoperatively, staged STABILISE-TEVAR was performed. Early staged STABILISE-TEVAR was technically feasible and achieved favorable aortic remodeling at 12 months in this patient.

## Abbreviations

CA
: celiac artery
CPB
: cardiopulmonary bypass
CTA
: computed tomography angiography
FET
: frozen elephant trunk
FL
: false lumen
SMA
: superior mesenteric artery
STABILISE
: stent-assisted balloon-induced intimal disruption and relamination in aortic dissection repair
TARFET
: total arch replacement with frozen elephant trunk
TEVAR
: thoracic endovascular aortic repair
TL
: true lumen

## Introduction

Acute type A aortic dissection carries high early mortality. Despite successful central repair, the descending false lumen (FL) remains patent in 50%–70% of patients.^1)^ Persistent FL patency is associated with distal aortic enlargement and late aortic events, although its effect on long-term mortality remains controversial. While total arch replacement with frozen elephant trunk (TARFET) promotes favorable aortic remodeling,^2)^ residual distal dissection often persists, requiring long-term surveillance and occasional secondary intervention.^3)^ In patients with residual distal dissection after central repair, thoracic endovascular aortic repair (TEVAR) alone is often insufficient to achieve complete remodeling in chronic dissection because of fibrosis and reduced intimal flap mobility.^4)^ Although acute or subacute TEVAR is associated with better remodeling than chronic intervention,^5)^ whether it alone achieves complete aortic remodeling remains uncertain.

First described by Hofferberth et al. in 2014, the stent-assisted balloon-induced intimal disruption and relamination in aortic dissection repair (STABILISE) technique combines a covered stent graft, bare-metal stents, and balloon remodeling to reappose the intimal flap to the aortic wall. This technique eliminates FL perfusion, creates a single aortic lumen, and promotes thoracoabdominal aortic remodeling.^6)^ However, experience with STABILISE during the subacute phase after central repair for an acute type A aortic dissection remains limited, and no detailed case reports have been published. Moreover, concerns regarding aortic rupture during balloon remodeling remain a major barrier to the widespread adoption of the STABILISE technique.

Here, we report successful staged STABILISE-TEVAR performed 38 days after TARFET for an acute type A aortic dissection, with favorable aortic remodeling at 12 months.

## Case Report

A 43-year-old male with hypertension presented with sudden-onset back pain followed by loss of consciousness. On arrival, he was diaphoretic, with a Glasgow Coma Scale score of 13 (E3V4M6), a systolic blood pressure of approximately 70 mmHg and an unmeasurable diastolic pressure, a heart rate of 115 beats/min, and an oxygen saturation of 90% while receiving 15 L/min of oxygen via a non-rebreather mask. Emergency transthoracic echocardiography revealed cardiac tamponade and an intimal flap in the ascending aorta. Electrocardiography showed ischemic changes in the right coronary artery territory. Computed tomography angiography (CTA) showed an acute type A aortic dissection with a primary tear in the ascending aorta, extending to both iliac arteries. The right coronary artery was avulsed. The celiac artery (CA), superior mesenteric artery (SMA), and right renal artery arose from the true lumen (TL); the left renal artery arose from the FL. Transesophageal echocardiography showed no aortic regurgitation and a primary tear in the ascending aorta.

Emergency surgery was performed for an acute type A aortic dissection complicated by cardiac tamponade and right coronary artery malperfusion. Given the young age of the patient and the extensive aortic dissection extending to both iliac arteries, a TARFET was performed to promote favorable aortic remodeling following central repair. The patient underwent emergency central repair through median sternotomy with cardiopulmonary bypass (CPB). A 9-mm J graft (Japan Lifeline, Tokyo, Japan) was anastomosed end-to-side to the left axillary artery for arterial cannulation. Venous cannulation was performed through the right atrium. The patient was cooled to 28°C. During cooling, concomitant right coronary artery bypass grafting using a saphenous vein graft was performed for the avulsed right coronary artery, and the cervical branches were dissected. After systemic circulatory arrest was established, the aorta was opened, and selective antegrade cerebral perfusion was initiated via the brachiocephalic and left common carotid arteries. After complete resection of the intimal tear, selective antegrade cardioplegia was administered directly into the left main coronary artery and through the saphenous vein graft to the right coronary artery. The aortic arch was transected between the origins of the left subclavian and left carotid arteries. A 27 × 60-mm FROZENIX FET graft (Japan Lifeline) was positioned into the TL. The distal aortic stump was reinforced with an external Teflon felt strip, while the proximal portion of the FET graft served as the internal reinforcement. Distal reconstruction was then completed using a 26-mm 4-branched J graft (Japan Lifeline), after which systemic circulation was re-established and rewarming was initiated. The proximal anastomosis was completed during rewarming. The aortic cross-clamp was removed after completion of the proximal anastomosis to restore coronary perfusion. The 9-mm J graft used for left axillary cannulation was routed through the left pleural cavity into the mediastinum and anastomosed to the left subclavian branch of the 4-branched graft. The left common carotid and brachiocephalic arteries were anastomosed to their respective graft branches. The operation, CPB, and circulatory arrest times were 408, 279, and 48 min, respectively. The patient was extubated on postoperative day 4, transferred from the intensive care unit on day 7, and discharged on day 20. Histopathologic examination of the resected aorta showed no evidence of an underlying connective tissue disorder. Serial CTA on postoperative days 7 and 17 demonstrated progressive changes. During the postoperative course, the patient developed postprandial abdominal pain and bilateral lower-extremity numbness. Therefore, repeat CTA was performed on postoperative day 17 to evaluate for possible new-onset malperfusion and to reassess the residual aortic dissection. Although no new malperfusion was identified, serial CTA demonstrated progressive FL enlargement with persistent TL compression. The maximum FL diameter increased from 23.0 to 26.0 mm at the descending thoracic aorta, from 22.5 to 25.2 mm at the CA level, and from 23.3 to 25.0 mm at the SMA level between postoperative day 7 and postoperative day 17, whereas the corresponding maximum TL diameters decreased from 22.6 to 21.6 mm, from 23.0 to 21.5 mm, and from 25.0 to 24.4 mm, respectively. The corresponding total aortic diameters changed from 26.5 to 27.1 mm, from 26.0 to 26.7 mm, and from 24.0 to 24.3 mm, respectively. Representative postoperative day 17 CTA images are shown in **Fig. 1**. CTA demonstrated persistent FL patency throughout the descending thoracoabdominal aorta (**Fig. 1A**), progressive FL enlargement (**Fig. 1B**), CA narrowing (**Fig. 1C**), persistent FL perfusion at the SMA (**Fig. 1D**), the right and left renal arteries arising from the true and FLs, respectively, with a re-entry tear at the left renal artery (**Fig. 1E** and **1F**), and marked infrarenal TL compression (**Fig. 1G**). Postoperative CTA demonstrated 7 distal re-entry tears beyond the FET: 2 in the descending thoracic aorta, including 1 approximately 4 cm distal to the distal edge of the FET; 1 large re-entry tear near the origin of the left renal artery; 2 in the infrarenal abdominal aorta; 1 in the left common iliac artery; and 1 in the right external iliac artery. Thus, multiple re-entry tears were present independently from the descending thoracic aorta to the iliac arteries. Among the visceral and renal branches, only the left renal artery originated from the FL. Given the persistent patency of the FL, progressive FL enlargement with TL compression, and the extensive residual dissection in this young patient, staged STABILISE-TEVAR was planned.

**Fig. 1:**
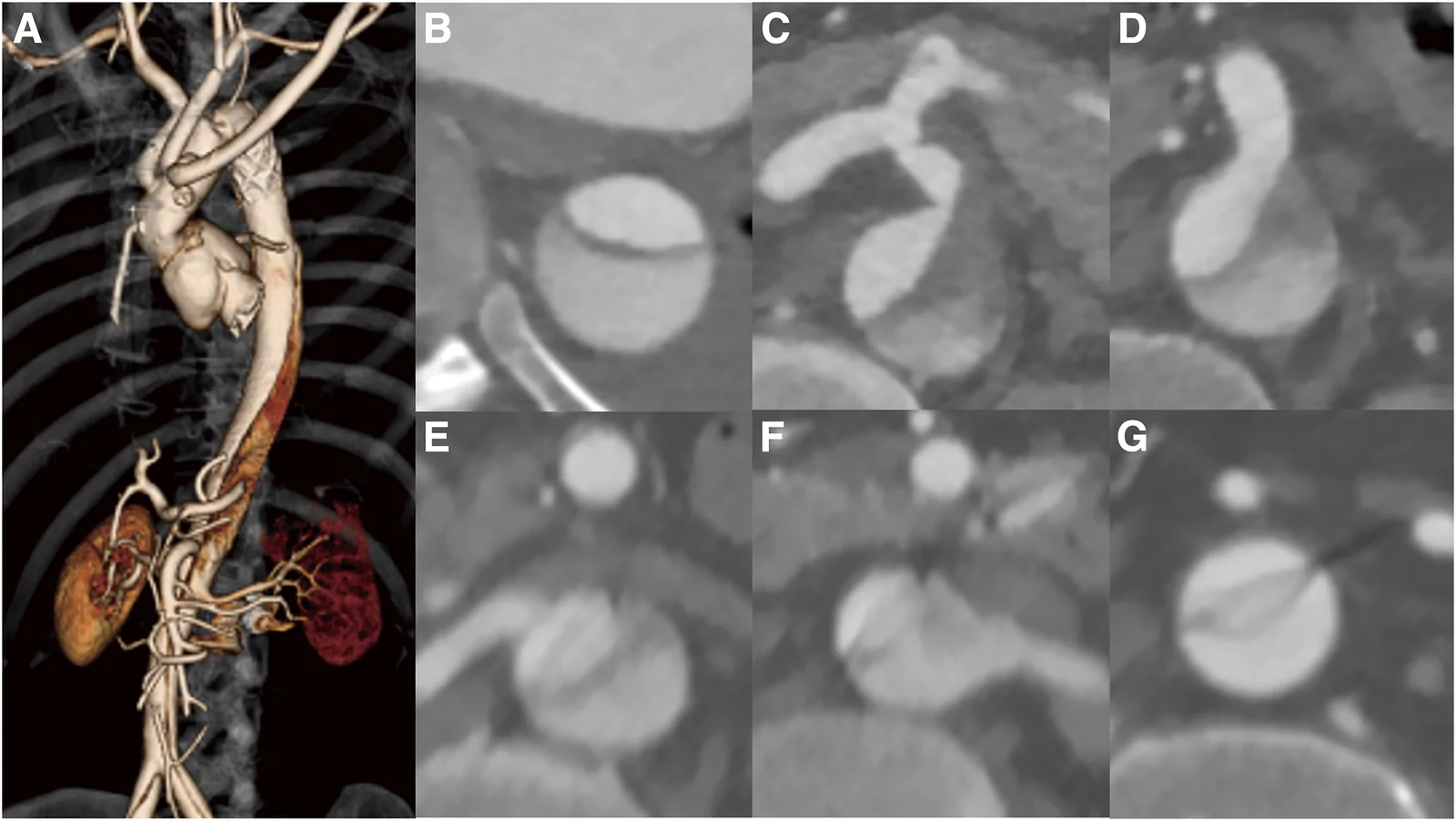
Contrast-enhanced computed tomography imaging after central repair. (**A**) Persistent false lumen patency throughout the descending thoracoabdominal aorta after total arch replacement with a frozen elephant trunk, with a patent saphenous vein graft to the right coronary artery. (**B**) False lumen enlargement in the proximal descending thoracic aorta at the level of the aortic valve. (**C**) Narrowing of the true lumen and the celiac artery origin. (**D**) Persistent false lumen perfusion at the level of the superior mesenteric artery origin. (**E**) Right renal artery arising from the true lumen. (**F**) Left renal artery arising from the false lumen. (**G**) True lumen compression in the infrarenal abdominal aorta.

Thirty-eight days after central repair, staged STABILISE-TEVAR was performed under general anesthesia (**Supplementary Video 1**). After TL catheterization using an extra-stiff guidewire (Lunderquist; Cook Medical, Bloomington, IN, USA), a thoracic stent graft (32-28-201, Zenith Alpha Thoracic; Cook Medical) was deployed, landing proximally within the FET and distally 7 cm proximal to the CA. Intraprocedural aortography demonstrated persistent re-entry flow into the FL at the descending thoracic aorta after stent graft deployment, confirming the indication for the STABILISE procedure (highlighted in **Supplementary Video 1**). Two overlapping bare-metal aortic stents (ZDES 36-123 and 36-164; Cook Medical) were sequentially deployed through the distal stent graft to 6 cm below the renal arteries in the infrarenal aorta, after which angiography demonstrated persistent false lumen opacification and a re-entry tear near the left renal artery origin (**Fig. 2A**). Finally, a Gekira Xb balloon catheter (Cosmotec, Tokyo, Japan) was used to initiate balloon remodeling at this pre-existing re-entry tear at the left renal artery origin (**Fig. 2B** and **2C**, white arrow). Sequential balloon dilatation was performed caudally to the distal end of the bare-metal stent in the infrarenal aorta and then continued cranially through the stents to promote FL obliteration (**Fig. 2D**–**2I**). No adjunctive visceral artery stenting was required. Completion angiography confirmed complete FL obliteration with preserved TL and visceral branch patency (**Fig. 2J**). The intraoperative angiography during the STABILISE-TEVAR procedure is shown in **Supplementary Video 1**. The patient was discharged on postoperative day 3. Pre-discharge CTA confirmed marked TL expansion, complete FL obliteration at the stented levels, and preserved visceral branch patency (**Fig. 3A**–**3G**). At 12 months, the patient remained asymptomatic and had resumed normal activities. Follow-up CTA demonstrated excellent aortic remodeling with sustained FL obliteration throughout the thoracoabdominal aorta, no aortic enlargement, and preserved visceral branch patency. No reintervention was required.

**Fig. 2:**
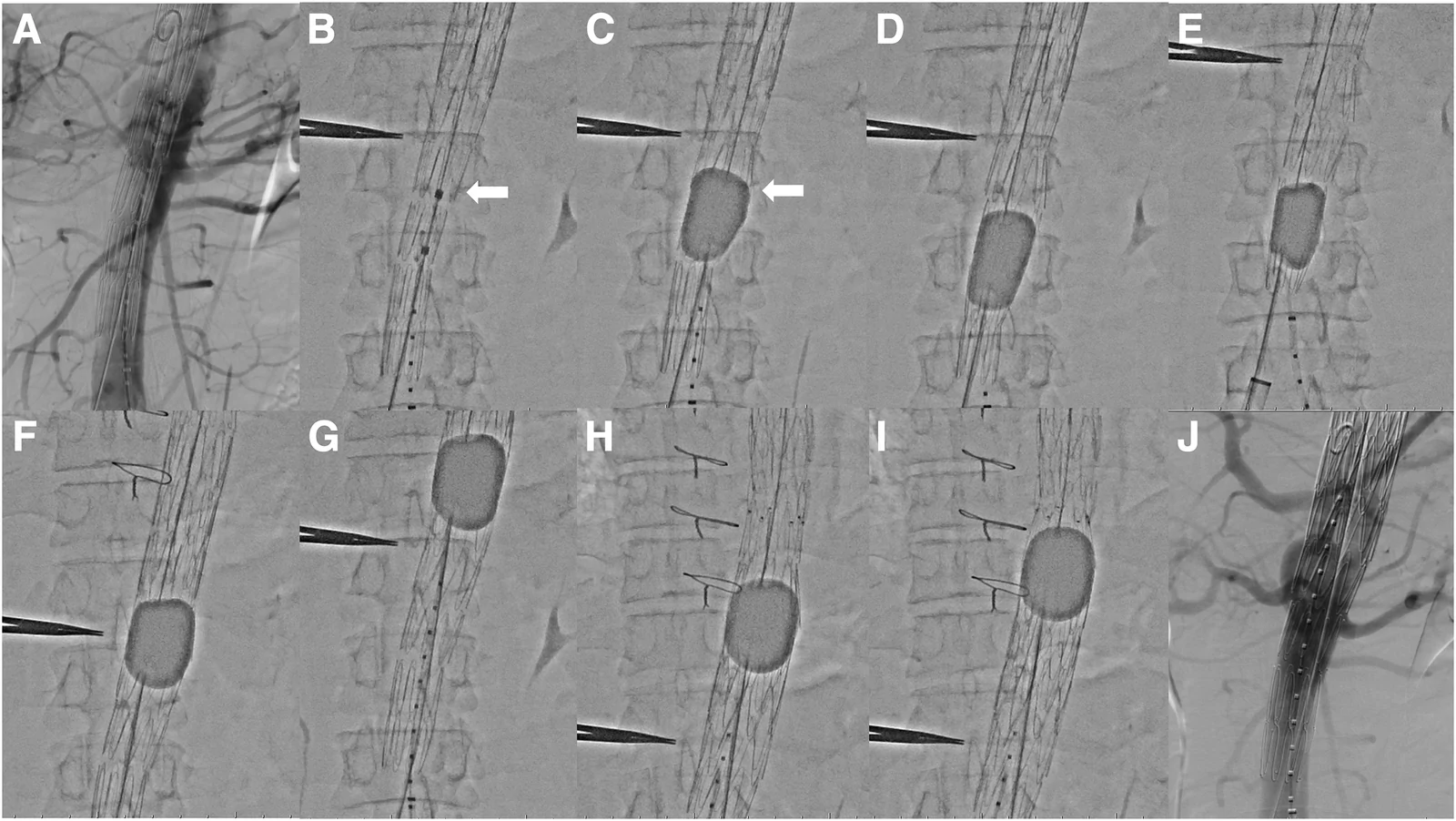
Angiography during STABILISE-TEVAR. (**A**) Angiography after bare-metal stent deployment showing persistent false lumen opacification and a re-entry tear near the origin of the left renal artery. (**B**) Before balloon remodeling with the STABILISE technique. The tip of a mosquito forceps was used as a fluoroscopic landmark to identify the level of the right renal artery and the re-entry tear at the origin of the left renal artery (white arrow). (**C**) After the initial STABILISE maneuver at the level of the re-entry tear (white arrow). (**D**) Subsequent caudal STABILISE maneuver. (**E**) Further caudal STABILISE maneuver near the distal end of the bare-metal stent. (**F**) Further cranial STABILISE maneuver near the renal arteries. (**G**) STABILISE maneuver at the suprarenal aortic level. (**H**, **I**) Further cranial STABILISE maneuvers. (**J**) Completion angiography confirming complete FL obliteration, TL patency, and patency of all visceral branches. FL, false lumen; TL, true lumen; STABILISE, stent-assisted balloon-induced intimal disruption and relamination in aortic dissection repair

**Fig. 3:**
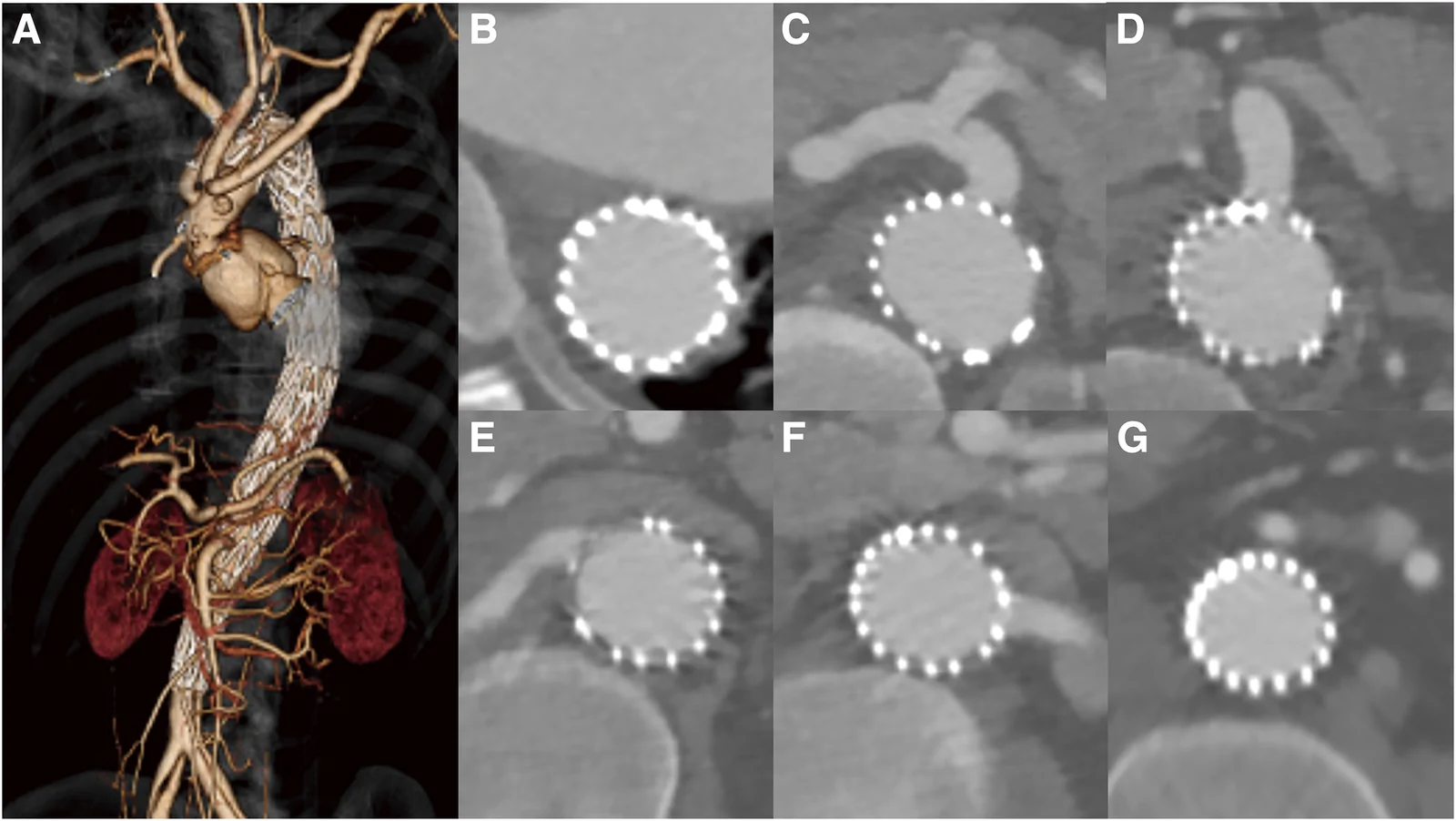
Contrast-enhanced computed tomography imaging after STABILISE-TEVAR. (**A**) True lumen expansion, complete false lumen obliteration at the stented levels, and preserved patency of all visceral branches. (**B**) Complete aortic remodeling at the descending aorta level. (**C**) Complete aortic remodeling at the celiac artery level. (**D**) Complete aortic remodeling at the superior mesenteric artery level. (**E**) Complete aortic remodeling at the right renal artery level. (**F**) Complete aortic remodeling at the left renal artery level. (**G**) Complete false lumen obliteration at the infrarenal abdominal aortic stented levels. STABILISE-TEVAR, stent-assisted balloon-induced intimal disruption and relamination in aortic dissection repair-thoracic endovascular aortic repair

## Discussion

This case demonstrates favorable remodeling after staged STABILISE-TEVAR following TARFET for an acute type A aortic dissection. As shown in this case, favorable aortic remodeling is not always achieved after TARFET. In such cases, additional stent-graft coverage may be a reasonable and less complex option when persistent FL perfusion is predominantly maintained by a focal re-entry tear that can be safely covered. In the present case, additional TEVAR to cover the proximal re-entry tear located approximately 4 cm distal to the FET, followed by observation for subsequent remodeling, was considered. However, multiple distal re-entry tears extended from the descending thoracic aorta to the iliac arteries, and the largest re-entry tear was located near the origin of the left renal artery, which originated from the FL. Therefore, covering only the proximal re-entry tear was considered unlikely to achieve sufficient abdominal aortic remodeling because the large re-entry tear near the left renal artery and multiple distal communications would remain. We therefore selected STABILISE to promote remodeling throughout the extensively dissected distal aorta while preserving visceral and renal branch perfusion. In selected patients with extensive residual dissection and multiple distal re-entry tears, STABILISE may be an option when simple stent-graft extension is unlikely to achieve sufficient distal remodeling; however, further evidence is needed to establish its role relative to alternative endovascular strategies. The STABILISE technique was introduced to promote aortic remodeling by creating a single-lumen aorta in patients with type B aortic dissection.^6,7)^ Although it was initially met with skepticism because of concerns about balloon expansion within the fragile, acutely dissected aorta, the STABILISE technique has demonstrated promising early outcomes with increasing clinical experience. Consequently, the technique is increasingly recognized as a method for promoting aortic remodeling in type B aortic dissection.^7)^ More recently, Faure et al. reported favorable aortic remodeling following STABILISE performed during the acute and subacute phases following central repair for an acute type A aortic dissection.^8)^ The same group subsequently reported satisfactory clinical and aortic remodeling outcomes after STABILISE for chronic residual distal dissection following central repair for an acute type A aortic dissection.^9)^ However, these findings were derived from a single center. Evidence supporting STABILISE after central repair for an acute type A aortic dissection remains limited, and detailed descriptions of procedural strategies and imaging findings are scarce.

A key aspect of our procedural strategy was initiating the STABILISE maneuver at a pre-existing re-entry tear. In this case, balloon remodeling was initiated at the large re-entry tear at the origin of the left renal artery, where the intimal flap was already disrupted (**Fig. 2B** and **2C** white arrow). We considered that initiating ballooning at this site may facilitate further intimal disruption without initially applying balloon expansion to an intact segment of the dissected flap. Balloon dilatation was subsequently extended caudally to the distal end of the bare-metal stent in the infrarenal aorta and then continued cranially through the stented segment to achieve FL obliteration (**Fig. 2D**–**2I**). However, this approach represents a technical consideration derived from this case rather than an established strategy. Whether initiating balloon remodeling at a pre-existing re-entry tear reduces the radial force required for intimal disruption or the risk of aortic rupture remains unknown and requires further investigation.

Aortic rupture is the most devastating complication of the STABILISE technique. Although rare, Hsu et al. reported a case of procedure-related aortic rupture that was successfully managed with prompt surgical intervention.^10)^ Balloon selection and inflation are therefore important technical considerations during STABILISE. In this case, the maximum total aortic diameters before STABILISE were 27.1 mm at the descending thoracic aorta, 26.7 mm at the CA level, 24.3 mm at the SMA level, 23.0 mm at the left renal artery level, 23.5 mm at the right renal artery level, and 22.0 mm in the infrarenal abdominal aorta. We selected the Gekira Xb balloon, which has a maximum diameter of 33 mm, smaller than the nominal diameter of the 36-mm bare-metal stent. Importantly, the balloon was not routinely expanded to its maximum diameter. Balloon inflation was performed gradually under fluoroscopic guidance and was terminated once flap disruption was suggested by tactile feedback from the balloon catheter and confirmed fluoroscopically. Thus, balloon expansion was limited to the minimum diameter necessary to achieve intimal disruption. This represents our technical approach to avoiding excessive balloon expansion; however, its effect on the risk of aortic rupture has not been established. Additionally, the timing of the STABILISE procedure may be critical for procedural safety. As the dissection progresses into the chronic phase, the intimal flap becomes progressively thicker and less compliant, requiring greater ballooning force to achieve intimal disruption, which may increase the risk of aortic rupture. Therefore, when the STABILISE technique is indicated, it should ideally be performed before the dissection reaches the chronic phase. Based on our experience, the acute, subacute, and possibly early chronic phases appear to provide the safest window for this technique, although further clinical evidence is needed to define the optimal timing.

## Conclusion

Early staged STABILISE-TEVAR was technically feasible and achieved favorable aortic remodeling at 12 months in this patient.

## Supplementary Materials

Supplementary Video 1 This video demonstrates the key steps of the STABILISE-TEVAR procedure. A thoracic stent graft and two overlapping bare-metal aortic stents were deployed from the frozen elephant trunk to the infrarenal aorta. Balloon remodeling was initiated at the re-entry tear near the origin of the left renal artery and performed sequentially in the caudal and cranial directions. Completion angiography confirmed complete false lumen obliteration with preservation of the true lumen and visceral branch patency.

## Declarations

### Data availability

All data generated or analyzed during this study are included in this published article.
